# Crystal structure of diethyl (*E*)-2-[(benzo­furan-2-yl)methyl­idene]succinate

**DOI:** 10.1107/S2056989015019313

**Published:** 2015-10-24

**Authors:** Marie-Luis Schirmer, Anke Spannenberg, Thomas Werner

**Affiliations:** a Leibniz-Institut für Katalyse e. V. an der Universität Rostock, Albert-Einstein-Strasse 29a, 18059 Rostock, Germany

**Keywords:** crystal structure, benzo­furan, diene, Wittig reaction, hydrogen bonding

## Abstract

The title compound, C_17_H_18_O_5_, was synthesized by a base-free catalytic Wittig reaction. The mol­ecule consists of a diethyl itaconate unit, which is connected *via* the C=C double bond to a benzo­furan moiety. The benzo­furan ring system (r.m.s. deviation = 0.007 Å) forms dihedral angles of 79.58 (4) and 12.12 (10)° with the mean planes through the *cis* and *trans* eth­oxy­carbonyl groups, respectively. An intra­molecular C—H⋯O hydrogen bond involving the O atom of the benzo­furan moiety is observed. In the crystal, mol­ecules are linked into ribbons running parallel to the *b* axis by C—H⋯O hydrogen bonds.

## Related literature   

For the synthesis of the title compound and related structures, see: Schirmer *et al.* (2015 ▸). For related crystal structures of similar compounds corresponding to (benzo­furan)-CH=C*R* ^1^ *R* ^2^, which only differ in *R* ^1^ and *R* ^2^ with at least one electron-withdrawing group, see: Penthala *et al.* (2012 ▸); Wei *et al.* (2011 ▸).
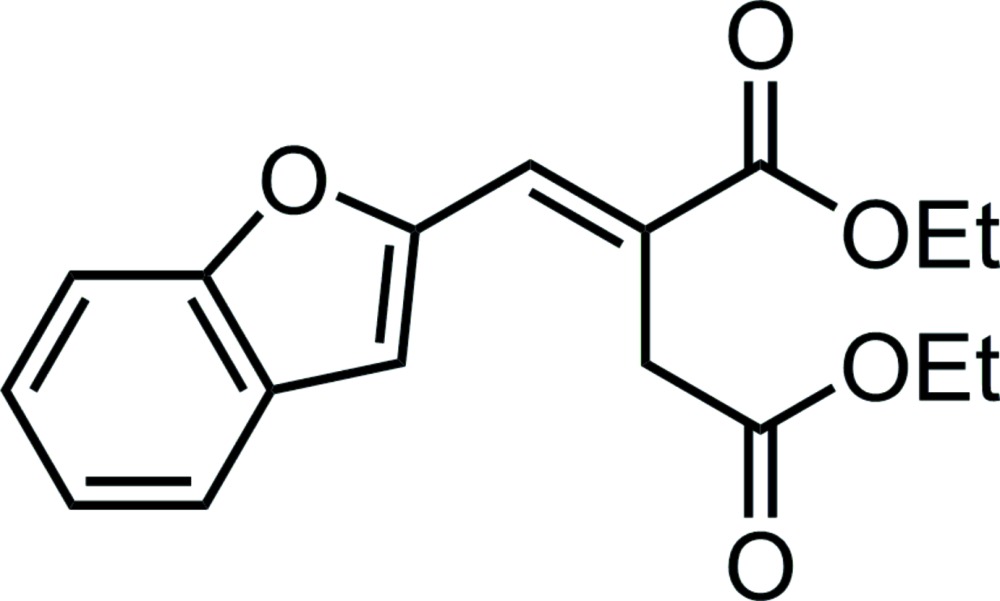


## Experimental   

### Crystal data  

C_17_H_18_O_5_
*M* *_r_* = 302.31
Orthorhombic, 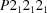

*a* = 7.0974 (4) Å
*b* = 8.0898 (4) Å
*c* = 26.9429 (15) Å
*V* = 1546.97 (14) Å^3^
*Z* = 4
Mo *K*α radiation
μ = 0.10 mm^−1^
*T* = 150 K
0.46 × 0.35 × 0.20 mm

### Data collection  

Bruker APEXII CCD diffractometer
Absorption correction: multi-scan (*SADABS*; Bruker, 2014 ▸) *T*_min_ = 0.84, *T*_max_ = 0.98
11102 measured reflections
4020 independent reflections
3645 reflections with *I* > 2σ(*I*)
*R*_int_ = 0.021

### Refinement  

*R*[*F* ^2^ > 2σ(*F* ^2^)] = 0.035
*wR*(*F* ^2^) = 0.087
*S* = 1.03
4020 reflections
201 parameters
H-atom parameters constrained
Δρ_max_ = 0.25 e Å^−3^
Δρ_min_ = −0.16 e Å^−3^

### 

Data collection: *APEX2* (Bruker, 2014 ▸); cell refinement: *SAINT* (Bruker, 2013 ▸); data reduction: *SAINT*; program(s) used to solve structure: *SHELXS97* (Sheldrick, 2008 ▸); program(s) used to refine structure: *SHELXL2014* (Sheldrick, 2015 ▸); molecular graphics: *XP* in *SHELXTL* (Sheldrick, 2008 ▸); software used to prepare material for publication: *SHELXTL*.

## Supplementary Material

Crystal structure: contains datablock(s) I, New_Global_Publ_Block. DOI: 10.1107/S2056989015019313/rz5171sup1.cif

Structure factors: contains datablock(s) I. DOI: 10.1107/S2056989015019313/rz5171Isup2.hkl

Supporting information file. DOI: 10.1107/S2056989015019313/rz5171Isup3.cml

**Fig. 1.**
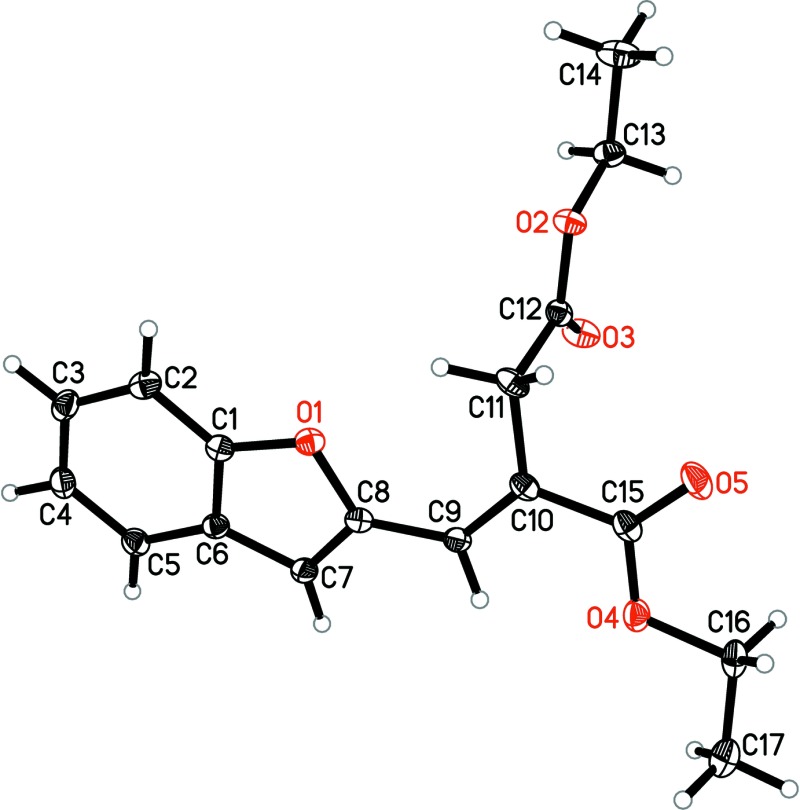
The molecular structure of the title compound, showing the atom labelling and displacement ellipsoids drawn at 30% probability level.

. DOI: 10.1107/S2056989015019313/rz5171fig1.tif
The mol­ecular structure of the title compound, showing the atom labelling and displacement ellipsoids drawn at 30% probability level.

CCDC reference: 1430813

Additional supporting information:  crystallographic information; 3D view; checkCIF report

## Figures and Tables

**Table 1:** Hydrogen-bond geometry (, )

*D*H*A*	*D*H	H*A*	*D* *A*	*D*H*A*
C2H2O3^i^	0.95	2.47	3.412(2)	170
C11H11*A*O5^ii^	0.99	2.52	3.458(2)	158
C11H11*B*O1	0.99	2.30	3.044(2)	131

Symmetry codes: (i) 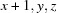; (ii) 
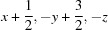
.

## supplementary crystallographic information

### S1. Synthesis and crystallization

Di­ethyl (*E*)-2-(benzo­furan-2-yl­methyl­ene) succinate was prepared as previously described by Schirmer *et al.* (2015). All reagents were purchased from commercial sources and used as received without further purification. The reaction was performed in 5 mL Wheaton screw-top V-Vials® with solid-top cap. The vial was dried in an oven at 120°C before use. Toluene was freshly distilled from sodium/benzo­phenone. Thin layer chromatography was performed on Merck TLC-plates with fluorescence indication (silica type 60, F254), spots were visualized using UV-light. Flash chromatography was performed using silica with a grain size of 40–63 µm from Macherey-Nagel. Benzo­furan-2-carbaldehyde (151 mg, 1.03 mmol) was dissolved in toluene (2 mL). Di­ethyl maleate (197 mg, 1.14 mmol), tri-*n*-butyl­phosphine (11 mg, 0.054 mmol) and phenyl­silane (113 mg, 1.04 mmol) were added successively to the solution. After stirring for 24 h at 125°C, the reaction mixture was cooled to ambient temperature. The crude product was purified by column chromatography over silica (SiO_2_) with cyclo­hexane/ethyl acetate (20:1 *v*/*v*) as eluents. All residues and volatiles were removed in vacuum (≤1 mbar, 60°C) to afford the olefination product (219 mg, 0.724 mmol, yield 70%, *E/Z* = 95:5) as colourless solid. Single crystals could be obtained by slow evaporation of a petroleum/di­chloro­methane (4:1 *v*/*v*) solution.

### S2. Refinement

H atoms were placed in idealized positions with d(C—H) = 0.95 Å (CH), 0.99 Å (CH_2_), 0.98 Å (CH_3_) and refined using a riding model with *U*_iso_(H) fixed at 1.2 *U*_eq_(C) for CH and CH_2_ and 1.5 *U*_eq_(C) for CH_3_. A rotating model was used for the methyl groups.

### Crystal data

**Table:**

C_17_H_18_O_5_	*D*_x_ = 1.298 Mg m^−^^3^
*M**_r_* = 302.31	Mo *K*α radiation, λ = 0.71073 Å
Orthorhombic, *P*2_1_2_1_2_1_	Cell parameters from 4958 reflections
*a* = 7.0974 (4) Å	θ = 2.6–28.6°
*b* = 8.0898 (4) Å	µ = 0.10 mm^−^^1^
*c* = 26.9429 (15) Å	*T* = 150 K
*V* = 1546.97 (14) Å^3^	Prism, colourless
*Z* = 4	0.46 × 0.35 × 0.20 mm
*F*(000) = 640	

### Data collection

**Table:**

Bruker APEXII CCD diffractometer	4020 independent reflections
Radiation source: fine-focus sealed tube	3645 reflections with *I* > 2σ(*I*)
Detector resolution: 8.3333 pixels mm^-1^	*R*_int_ = 0.021
φ and ω scans	θ_max_ = 28.8°, θ_min_ = 1.5°
Absorption correction: multi-scan (*SADABS*; Bruker, 2014)	*h* = −9→8
*T*_min_ = 0.84, *T*_max_ = 0.98	*k* = −9→10
11102 measured reflections	*l* = −35→36

### Refinement

**Table:**

Refinement on *F*^2^	Hydrogen site location: inferred from neighbouring sites
Least-squares matrix: full	H-atom parameters constrained
*R*[*F*^2^ > 2σ(*F*^2^)] = 0.035	*w* = 1/[σ^2^(*F*_o_^2^) + (0.0447*P*)^2^ + 0.2481*P*] where *P* = (*F*_o_^2^ + 2*F*_c_^2^)/3
*wR*(*F*^2^) = 0.087	(Δ/σ)_max_ < 0.001
*S* = 1.03	Δρ_max_ = 0.25 e Å^−^^3^
4020 reflections	Δρ_min_ = −0.16 e Å^−^^3^
201 parameters	Absolute structure: Flack *x* determined using 1441 quotients [(*I*^+^)-(*I*^-^)]/[(*I*^+^)+(*I*^-^)] (Parsons *et al.*, 2013)
0 restraints	Absolute structure parameter: −0.4 (3)

### Special details

**Table:**

Geometry. All e.s.d.'s (except the e.s.d. in the dihedral angle between two l.s. planes) are estimated using the full covariance matrix. The cell e.s.d.'s are taken into account individually in the estimation of e.s.d.'s in distances, angles and torsion angles; correlations between e.s.d.'s in cell parameters are only used when they are defined by crystal symmetry. An approximate (isotropic) treatment of cell e.s.d.'s is used for estimating e.s.d.'s involving l.s. planes.

### Fractional atomic coordinates and isotropic or equivalent isotropic displacement parameters (Å^2^)

**Table:**

	*x*	*y*	*z*	*U*_iso_*/*U*_eq_	
C1	1.1845 (2)	0.8258 (2)	0.20175 (6)	0.0226 (3)	
C2	1.3594 (3)	0.9003 (2)	0.20151 (7)	0.0283 (4)	
H2	1.4074	0.9551	0.1730	0.034*	
C3	1.4612 (3)	0.8902 (2)	0.24552 (8)	0.0317 (4)	
H3	1.5820	0.9403	0.2473	0.038*	
C4	1.3901 (3)	0.8079 (2)	0.28718 (7)	0.0308 (4)	
H4	1.4637	0.8033	0.3166	0.037*	
C5	1.2152 (3)	0.7334 (2)	0.28647 (6)	0.0282 (4)	
H5	1.1677	0.6777	0.3149	0.034*	
C6	1.1097 (2)	0.7422 (2)	0.24256 (6)	0.0235 (3)	
C7	0.9292 (3)	0.6833 (2)	0.22697 (6)	0.0251 (3)	
H7	0.8431	0.6206	0.2463	0.030*	
C8	0.9040 (3)	0.7338 (2)	0.17946 (6)	0.0237 (3)	
C9	0.7443 (3)	0.7013 (2)	0.14801 (6)	0.0231 (3)	
H9	0.6562	0.6249	0.1613	0.028*	
C10	0.6987 (3)	0.7607 (2)	0.10299 (6)	0.0238 (3)	
C11	0.8049 (3)	0.8887 (2)	0.07369 (6)	0.0266 (4)	
H11A	0.8143	0.8530	0.0386	0.032*	
H11B	0.9342	0.8994	0.0871	0.032*	
C12	0.7066 (3)	1.0538 (2)	0.07631 (6)	0.0221 (3)	
C13	0.6907 (3)	1.3237 (2)	0.04296 (7)	0.0275 (4)	
H13A	0.7166	1.3773	0.0753	0.033*	
H13B	0.5525	1.3171	0.0384	0.033*	
C14	0.7776 (4)	1.4218 (3)	0.00172 (8)	0.0411 (5)	
H14A	0.9137	1.4307	0.0072	0.062*	
H14B	0.7219	1.5326	0.0010	0.062*	
H14C	0.7541	1.3662	−0.0300	0.062*	
C15	0.5193 (3)	0.7059 (2)	0.07940 (6)	0.0264 (4)	
C16	0.2480 (3)	0.5350 (3)	0.08479 (7)	0.0333 (4)	
H16A	0.2709	0.4904	0.0511	0.040*	
H16B	0.1552	0.6259	0.0823	0.040*	
C17	0.1757 (3)	0.4019 (3)	0.11828 (9)	0.0423 (5)	
H17A	0.2665	0.3106	0.1193	0.063*	
H17B	0.0547	0.3612	0.1057	0.063*	
H17C	0.1586	0.4465	0.1518	0.063*	
O1	1.05847 (18)	0.82331 (16)	0.16268 (4)	0.0251 (3)	
O2	0.77290 (18)	1.15850 (15)	0.04184 (4)	0.0255 (3)	
O3	0.5870 (2)	1.09132 (17)	0.10586 (5)	0.0325 (3)	
O4	0.42296 (19)	0.59499 (17)	0.10627 (5)	0.0294 (3)	
O5	0.4678 (2)	0.75572 (19)	0.03924 (5)	0.0382 (4)	

### Atomic displacement parameters (Å^2^)

**Table:**

	*U*^11^	*U*^22^	*U*^33^	*U*^12^	*U*^13^	*U*^23^
C1	0.0260 (8)	0.0202 (8)	0.0217 (7)	0.0027 (7)	0.0028 (6)	−0.0030 (6)
C2	0.0274 (9)	0.0263 (9)	0.0314 (9)	−0.0004 (8)	0.0098 (7)	−0.0024 (7)
C3	0.0213 (8)	0.0279 (9)	0.0460 (10)	0.0003 (8)	0.0010 (8)	−0.0071 (8)
C4	0.0287 (9)	0.0290 (9)	0.0349 (9)	0.0047 (8)	−0.0086 (7)	−0.0045 (8)
C5	0.0316 (9)	0.0282 (9)	0.0247 (8)	0.0011 (8)	−0.0019 (7)	0.0020 (7)
C6	0.0244 (8)	0.0210 (8)	0.0250 (8)	0.0004 (7)	0.0018 (6)	−0.0007 (7)
C7	0.0263 (8)	0.0256 (8)	0.0234 (7)	−0.0035 (8)	−0.0011 (6)	0.0041 (7)
C8	0.0273 (9)	0.0205 (8)	0.0232 (7)	−0.0006 (7)	0.0013 (6)	0.0011 (6)
C9	0.0272 (8)	0.0196 (8)	0.0227 (7)	−0.0003 (7)	−0.0006 (6)	0.0014 (6)
C10	0.0307 (9)	0.0203 (8)	0.0205 (7)	0.0061 (7)	0.0005 (6)	−0.0011 (6)
C11	0.0345 (9)	0.0251 (9)	0.0202 (7)	0.0088 (8)	0.0052 (7)	0.0034 (7)
C12	0.0271 (9)	0.0218 (8)	0.0176 (7)	0.0003 (7)	−0.0019 (6)	−0.0007 (6)
C13	0.0286 (9)	0.0196 (8)	0.0343 (9)	0.0029 (7)	0.0057 (7)	0.0000 (7)
C14	0.0496 (13)	0.0254 (10)	0.0483 (12)	0.0056 (10)	0.0184 (10)	0.0096 (9)
C15	0.0338 (9)	0.0224 (9)	0.0230 (8)	0.0098 (7)	−0.0032 (7)	−0.0045 (6)
C16	0.0259 (9)	0.0382 (11)	0.0356 (10)	0.0064 (9)	−0.0093 (8)	−0.0103 (8)
C17	0.0294 (10)	0.0500 (14)	0.0475 (12)	−0.0045 (10)	−0.0053 (9)	−0.0038 (10)
O1	0.0295 (6)	0.0262 (6)	0.0197 (5)	−0.0021 (6)	0.0032 (5)	0.0010 (5)
O2	0.0296 (7)	0.0208 (6)	0.0261 (6)	0.0035 (5)	0.0068 (5)	0.0031 (5)
O3	0.0433 (8)	0.0269 (7)	0.0272 (6)	0.0057 (6)	0.0126 (6)	0.0008 (5)
O4	0.0296 (7)	0.0320 (7)	0.0266 (6)	0.0006 (6)	−0.0073 (5)	−0.0028 (5)
O5	0.0503 (9)	0.0375 (8)	0.0267 (6)	0.0075 (7)	−0.0135 (6)	0.0018 (6)

### Geometric parameters (Å, º)

**Table:**

C1—C2	1.380 (3)	C11—H11A	0.9900
C1—O1	1.381 (2)	C11—H11B	0.9900
C1—C6	1.396 (2)	C12—O3	1.203 (2)
C2—C3	1.391 (3)	C12—O2	1.342 (2)
C2—H2	0.9500	C13—O2	1.459 (2)
C3—C4	1.399 (3)	C13—C14	1.498 (3)
C3—H3	0.9500	C13—H13A	0.9900
C4—C5	1.380 (3)	C13—H13B	0.9900
C4—H4	0.9500	C14—H14A	0.9800
C5—C6	1.402 (2)	C14—H14B	0.9800
C5—H5	0.9500	C14—H14C	0.9800
C6—C7	1.430 (2)	C15—O5	1.211 (2)
C7—C8	1.355 (2)	C15—O4	1.340 (2)
C7—H7	0.9500	C16—O4	1.453 (2)
C8—O1	1.389 (2)	C16—C17	1.496 (3)
C8—C9	1.439 (2)	C16—H16A	0.9900
C9—C10	1.344 (2)	C16—H16B	0.9900
C9—H9	0.9500	C17—H17A	0.9800
C10—C15	1.491 (3)	C17—H17B	0.9800
C10—C11	1.504 (3)	C17—H17C	0.9800
C11—C12	1.509 (2)		
			
C2—C1—O1	125.84 (16)	H11A—C11—H11B	108.1
C2—C1—C6	123.88 (16)	O3—C12—O2	123.10 (16)
O1—C1—C6	110.28 (15)	O3—C12—C11	125.51 (16)
C1—C2—C3	115.95 (17)	O2—C12—C11	111.37 (14)
C1—C2—H2	122.0	O2—C13—C14	107.76 (15)
C3—C2—H2	122.0	O2—C13—H13A	110.2
C2—C3—C4	121.64 (17)	C14—C13—H13A	110.2
C2—C3—H3	119.2	O2—C13—H13B	110.2
C4—C3—H3	119.2	C14—C13—H13B	110.2
C5—C4—C3	121.42 (17)	H13A—C13—H13B	108.5
C5—C4—H4	119.3	C13—C14—H14A	109.5
C3—C4—H4	119.3	C13—C14—H14B	109.5
C4—C5—C6	118.00 (17)	H14A—C14—H14B	109.5
C4—C5—H5	121.0	C13—C14—H14C	109.5
C6—C5—H5	121.0	H14A—C14—H14C	109.5
C1—C6—C5	119.10 (16)	H14B—C14—H14C	109.5
C1—C6—C7	105.72 (15)	O5—C15—O4	123.46 (18)
C5—C6—C7	135.18 (16)	O5—C15—C10	122.67 (18)
C8—C7—C6	107.16 (16)	O4—C15—C10	113.86 (15)
C8—C7—H7	126.4	O4—C16—C17	107.07 (16)
C6—C7—H7	126.4	O4—C16—H16A	110.3
C7—C8—O1	111.12 (16)	C17—C16—H16A	110.3
C7—C8—C9	127.21 (17)	O4—C16—H16B	110.3
O1—C8—C9	121.66 (14)	C17—C16—H16B	110.3
C10—C9—C8	130.98 (17)	H16A—C16—H16B	108.6
C10—C9—H9	114.5	C16—C17—H17A	109.5
C8—C9—H9	114.5	C16—C17—H17B	109.5
C9—C10—C15	118.98 (17)	H17A—C17—H17B	109.5
C9—C10—C11	126.78 (17)	C16—C17—H17C	109.5
C15—C10—C11	114.12 (15)	H17A—C17—H17C	109.5
C10—C11—C12	110.69 (15)	H17B—C17—H17C	109.5
C10—C11—H11A	109.5	C1—O1—C8	105.71 (13)
C12—C11—H11A	109.5	C12—O2—C13	115.07 (13)
C10—C11—H11B	109.5	C15—O4—C16	116.38 (14)
C12—C11—H11B	109.5		
			
O1—C1—C2—C3	178.71 (17)	C9—C10—C11—C12	−103.4 (2)
C6—C1—C2—C3	−0.8 (3)	C15—C10—C11—C12	72.54 (18)
C1—C2—C3—C4	0.5 (3)	C10—C11—C12—O3	15.9 (3)
C2—C3—C4—C5	−0.1 (3)	C10—C11—C12—O2	−165.74 (14)
C3—C4—C5—C6	0.0 (3)	C9—C10—C15—O5	178.99 (17)
C2—C1—C6—C5	0.7 (3)	C11—C10—C15—O5	2.7 (2)
O1—C1—C6—C5	−178.89 (15)	C9—C10—C15—O4	−2.1 (2)
C2—C1—C6—C7	−179.37 (17)	C11—C10—C15—O4	−178.38 (15)
O1—C1—C6—C7	1.0 (2)	C2—C1—O1—C8	179.37 (17)
C4—C5—C6—C1	−0.3 (3)	C6—C1—O1—C8	−1.04 (19)
C4—C5—C6—C7	179.9 (2)	C7—C8—O1—C1	0.65 (19)
C1—C6—C7—C8	−0.6 (2)	C9—C8—O1—C1	−178.34 (16)
C5—C6—C7—C8	179.3 (2)	O3—C12—O2—C13	0.7 (2)
C6—C7—C8—O1	0.0 (2)	C11—C12—O2—C13	−177.72 (14)
C6—C7—C8—C9	178.90 (17)	C14—C13—O2—C12	−178.92 (16)
C7—C8—C9—C10	171.75 (19)	O5—C15—O4—C16	−0.2 (3)
O1—C8—C9—C10	−9.4 (3)	C10—C15—O4—C16	−179.08 (14)
C8—C9—C10—C15	−178.91 (17)	C17—C16—O4—C15	174.43 (16)
C8—C9—C10—C11	−3.1 (3)		

### Hydrogen-bond geometry (Å, º)

**Table:**

*D*—H···*A*	*D*—H	H···*A*	*D*···*A*	*D*—H···*A*
C2—H2···O3^i^	0.95	2.47	3.412 (2)	170
C11—H11*A*···O5^ii^	0.99	2.52	3.458 (2)	158
C11—H11*B*···O1	0.99	2.30	3.044 (2)	131

Symmetry codes: (i) *x*+1, *y*, *z*; (ii) *x*+1/2, −*y*+3/2, −*z*.
