# Supplementary figures and images for: Crystal structure of diethyl (E)-2-[(benzo­furan-2-yl)methyl­idene]succinate
Source: Acta Crystallogr E Crystallogr Commun. 2015 Oct 24;71(Pt 11):o872. doi: 10.1107/S2056989015019313 (PMC4645082; doi:10.1107/S2056989015019313)

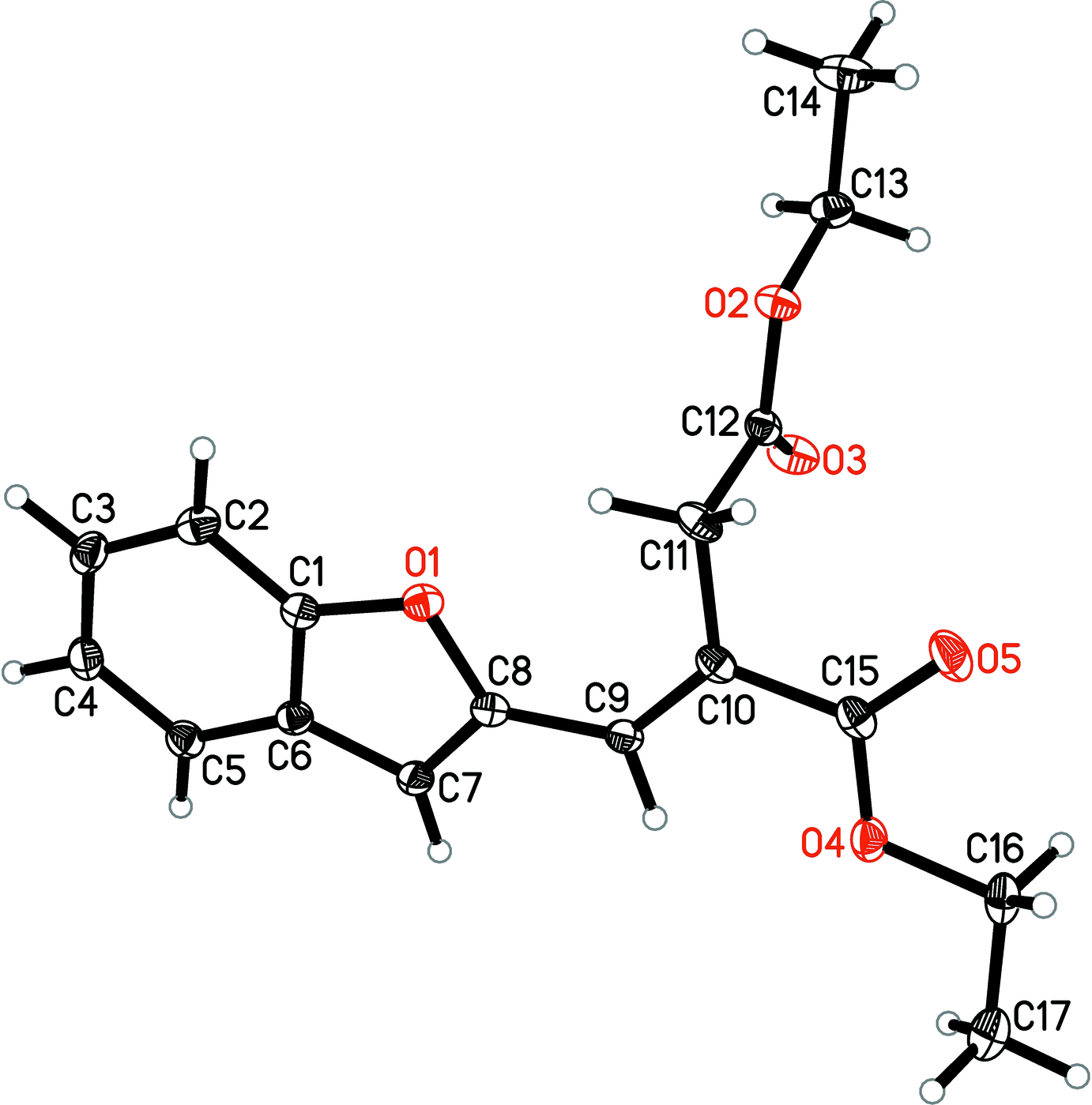

Supplement: Supplementary file 4 [file e-71-0o872-fig1.tif]
